# Emerging trends and hotspots in the links between the gut microbiota and MAFLD from 2002 to 2021: A bibliometric analysis

**DOI:** 10.3389/fendo.2022.990953

**Published:** 2022-10-13

**Authors:** Yixuan Li, Yanyu Zhou, Liya Wang, Xiaoqi Lin, Menghan Mao, Suqing Yin, Ling Zhu, Yingfu Jiao, Weifeng Yu, Po Gao, Liqun Yang

**Affiliations:** ^1^ Department of Anesthesiology, Renji Hospital, Shanghai Jiao Tong University School of Medicine, Shanghai, China; ^2^ Department of Gynecologic Oncology, International Peace Maternity and Child Health Hospital, Shanghai Jiao Tong University School of Medicine, Shanghai Municipal Key Clinical Specialty, Shanghai Key Laboratory of Embryo Original Disease, Shanghai, China

**Keywords:** gut microbiota (GM), MAFLD, bibliometric analysis (BA), hotspots, CiteSpace

## Abstract

**Background:**

The prevalence of metabolic associated fatty liver disease (MAFLD) presented a booming growth over recent years in the whole world. MAFLD was associated with a higher risk of end-stage liver disease, hepatocellular carcinoma and liver transplantation. Accumulating evidence indicated that gut microbiota and MAFLD were interrelated and interacted with each other. However, to the knowledge of the authors, no bibliometric quantitative analysis has been carried out to evaluate the links between the gut microbiota and MAFLD. This study aimed to use bibliometric analysis to evaluate current publication trends and hotspots in the links between the gut microbiota and MAFLD, in order to advance research in this field.

**Methods:**

The articles regarding the links between gut microbiota and MAFLD from 2002 to 2021 were identified from the Science Citation Index-Expanded of Web of Science Core Collection. CiteSpace software, Vosviewer, the R package “bibliometrix” and the Online Analysis Platform of Literature Metrology were used to analyze current publication trends and hotspots in this field.

**Results:**

A total of 707 articles were retrieved regarding the links between gut microbiota and MAFLD from 2002 to 2021. The USA occupied the leading role until 2015 and the dominance of China started in 2016. The USA was the most frequently involved country in international cooperation. Shanghai Jiao Tong University was the most productive institution. Ina Bergheim was the most productive author, publishing 14 articles. The co-citation keywords cluster label displayed ten main clusters: probiotics, bile acid, immune function, adolescents, nutritional genomics, high fat diet, systems biology, lipopolysaccharides, phosphatidylcholine, and oxidative stress. Keyword bursts analysis indicated that diet induced obesity, metabolic syndrome, ppar alpha, and lactobacillus were the research hotspots with high strength.

**Conclusion:**

The number of publications covering the links of gut microbiota and MAFLD increased dramatically in the past decade and especially became exponential growth in the last 3 years. Probiotics and bile acid will be the research direction of great importance in the etiology and novel treatment for MAFLD. This study provided systematic information and instructive assistance for future research work, that helped to discover the mechanisms and new treatments of MAFLD.

## Introduction

Metabolic associated fatty liver disease (MAFLD), a chronic hepatic disease, formerly named non-alcoholic fatty liver disease (NAFLD). The renaming from NAFLD to MAFLD in 2020 was due to the need for precise diagnosis and effective treatment (1). The diagnostic criteria of MAFLD requires the presence of metabolic risk factors in the setting of hepatic steatosis, includes individuals with other concomitant liver diseases, and excludes those with hepatic steatosis who do not fulfill the metabolic risk criteria (2). Following the transformation to a nutrient-abundant and sedentary lifestyle, the prevalence of MAFLD presented a booming growth which attracted doctors’ and researchers’ attention. MAFLD influenced almost 25% of adult people around the world, placing a significant burden on the health system and exacting a growing economic burden, but it had no targeted therapy so far (3). A subset of individuals with MAFLD may progress to nonalcoholic steatohepatitis (NASH), cirrhosis, and hepatocellular carcinoma (HCC) (4, 5). Several cohort studies have consistently demonstrated that MAFLD was associated with a higher risk of all-cause mortality and the leading cause of death in patients with MAFLD was cardiovascular disease (CVDs), which meant MAFLD was associated with an increased risk of major cardiovascular events and other cardiac complications (6). It has become clear that the net accumulation of lipid in the liver was the main characteristic of MAFLD (7). Hepatic lipid accumulation is induced by four separate mechanisms: (i) increased hepatic uptake of circulating fatty acids, (ii) increased hepatic *de novo* fatty acid synthesis, (iii) decreased hepatic beta-oxidation, and (iv) decreased hepatic lipid export (8). Moreover, recent work has produced these findings that gut microbiota also has an impact on the developmental trajectory of MAFLD (9).

Gut microbiota contains 10–100 trillion microorganisms, which have similar cell counts with human cells (10). These intestinal microbiotas thrive in a symbiotic fashion in the alimentary tract, communicating with host through metabolites, microbiota-associated molecular pattern (MAMP), membrane vesicles and other approaches (11, 12). Due to the widespread effect of microbial communities on the intestine and distal organs, microbiome research has expanded to nearly all systems in the human body over the past decades (13). For example, the microbiota-gut-brain (MGB) axis is a vital role in brain development, behavior, and function (14). Perturbations of the gut microbiota could lead to Alzheimer’s disease, anxiety, depression, and other brain-associated diseases (15). Gut-liver axis is another approach that has received increasing attention in the host-microbiota interaction. The embryological origin of the liver directly comes from the foregut during development, and the presence of the portal vein ensures the connection between microbiome and liver. This evidence includes the observations that intestinal permeability is increased in MAFLD patients compared with those without the disease and the effects of manipulation of the microflora on liver injury (16, 17). Recent work has provided clear evidence that dysbiosis promoted MAFLD through several mechanisms, including dysfunction of bile acid metabolism, reduced short-chain fatty acids (SCFA), inhibition of the production of fasting-induced adipocyte factor (FIAF), increasing intestinal permeability, and changes in gut motility (16). In this sense, researchers design “bacteriotherapy” as adjunctive treatments for MAFLD, such as probiotics, prebiotics, and symbiotics. It is reported that administration of a mixture of eight probiotic strains called VSL#3 to kids with MAFLD for 4 months decreased BMI and ultrasonographic steatosis through increasing circulating GLP-1 (18). In conclusion, disruption of gut microbiota is now a vital cause of MALFD and the development of MAFLD also changes the composition of the microbiota. Therefore, developing novel treatments for MAFLD targeted at microbiota will be a future research effort.

The growing prevalence of MAFLD and the hot trend in the research of gut microbiota brought the increased need for efforts on in-depth research in this field, but there are few articles summarizing the latest trend in this field and predicting research hotspots. Bibliometric analysis refers to a timely and comprehensive review of publications through the analysis of the number of publications, authors, countries and regions, references, keywords, and other parameters during a given period. It provides a detailed overview of the area of knowledge and keeps researchers abreast of the latest research trends (19). As far as we know, no bibliometric study has been reported to evaluate the hotspots and research trends on the links between gut microbiota and MAFLD. The aim of our study was to analyze publication trends about the connection between the gut microbiota and MAFLD from 2002-2021 and provide a panoramic vision and guidance for other researchers.

## Materials and methods

### Data sources and search strategies

We conducted a comprehensive literature search within the Science Citation Index-Expanded (SCI-E) of Web of Science Core Collection (WoSCC) database for the period 2002-2021 on March 28, 2022. Also, a literature search by PubMed database was conducted on September 25, 2022 for a supplement. In order to reduce the bias incurred by frequent database updates, we completed the search and retrieved data in 1 day.

**Figure 1:**
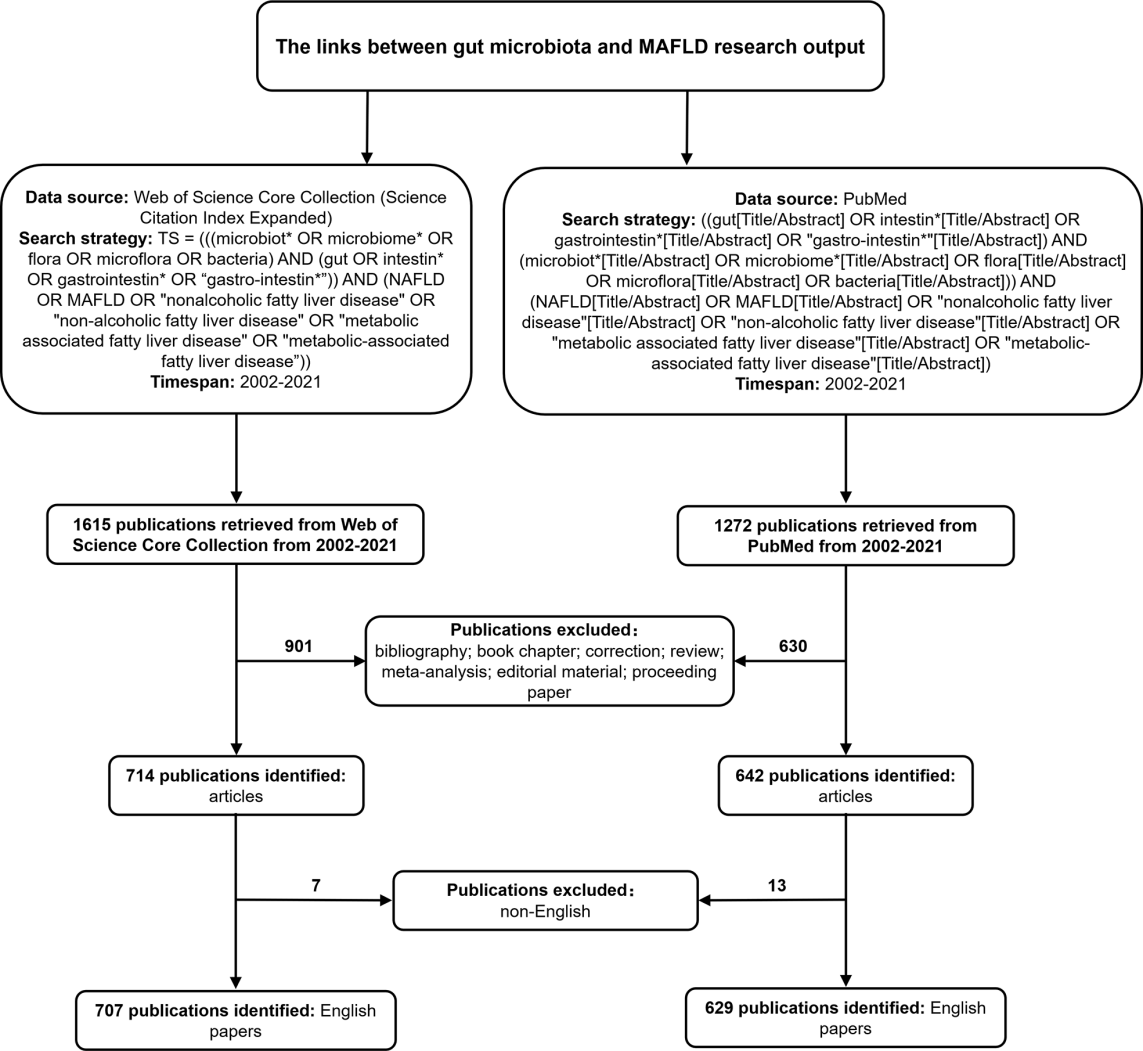
Flowchart for including and excluding literature studies.

We retrieved relevant publications in WoSCC through the following search strategy: TS = (((microbiot* OR microbiome* OR flora OR microflora OR bacteria) AND (gut OR intestin* OR gastrointestin* OR “gastro-intestin*”)) AND (NAFLD OR MAFLD OR “nonalcoholic fatty liver disease” OR “non-alcoholic fatty liver disease” OR “metabolic associated fatty liver disease” OR “metabolic-associated fatty liver disease”)) AND Language=English and Document type=Article. Only the SCI-E database was selected. The search strategy for PubMed was ((gut[Title/Abstract] OR intestin*[Title/Abstract] OR gastrointestin*[Title/Abstract] OR “gastro-intestin*”[Title/Abstract]) AND (microbiot*[Title/Abstract] OR microbiome*[Title/Abstract] OR flora[Title/Abstract] OR microflora[Title/Abstract] OR bacteria[Title/Abstract])) AND (NAFLD[Title/Abstract] OR MAFLD[Title/Abstract] OR “nonalcoholic fatty liver disease”[Title/Abstract] OR “non-alcoholic fatty liver disease”[Title/Abstract] OR “metabolic associated fatty liver disease”[Title/Abstract] OR “metabolic-associated fatty liver disease”[Title/Abstract]). The exclusion criteria were as follows: (i) no statements relating to the composition of the microbiota, (ii) no statement relating to MAFLD, (iii) only articles were included, bibliography, book chapter, correction, review, meta-analysis, editorial material, and proceeding paper were excluded and (iv) duplicate publication were excluded. To confirm the accuracy of bibliometric analysis results, the reviewer Yixuan Li identified all publications retrieved by the search strategy above, including titles, abstracts, and publication years. In total, the articles on the links between gut microbiota and MAFLD were ultimately analyzed in our study. The detailed screening is shown in **Figure 1**.

### Bibliometric analysis

In order to describe all literature characteristics about the connection between the gut microbiota and MAFLD research, we converted all data that met the requirements from WoSCC to TXT format and imported them into the Online Analysis Platform of Literature Metrology (https://bibliometric.com/app); Bibliometrix; CiteSpace V5.8 R3 (Drexel University, Philadelphia, PA, USA) and VOSviewer 1.6.15 (Leiden University, Leiden, The Nertherlands) for further analysis (20–22).

The annual publication number was from WoSCC and the research types from the data were selected by Yixuan Li. The publication number from the top 10 countries/regions and the top 10 most productive journals were exported from the Online Analysis Platform of Literature Metrology. The R package bibliometrix was used to output the top 100 high-frequency keywords as a word cloud. Moreover, the collaborations between countries/regions and those between institutions were analyzed by VOSviewer software. Article citations were from journal websites. As the most popular and recognized bibliometric visualization tool (23), CiteSpace was used to output various figures to help understand the recent status of the links between the gut microbiota and MAFLD research and to generate potential hotspots in this field, including the collaboration between authors, co-citation analysis, citation burst, clustered networks of co-cited references and keywords with the strongest citation bursts. For keywords burst detection, we deleted the ones with little real significance such as animal model, accumulation, weight loss, prevalence, and mice.

## Results

### Quantity and trends analysis of published papers

**Figure 2:**
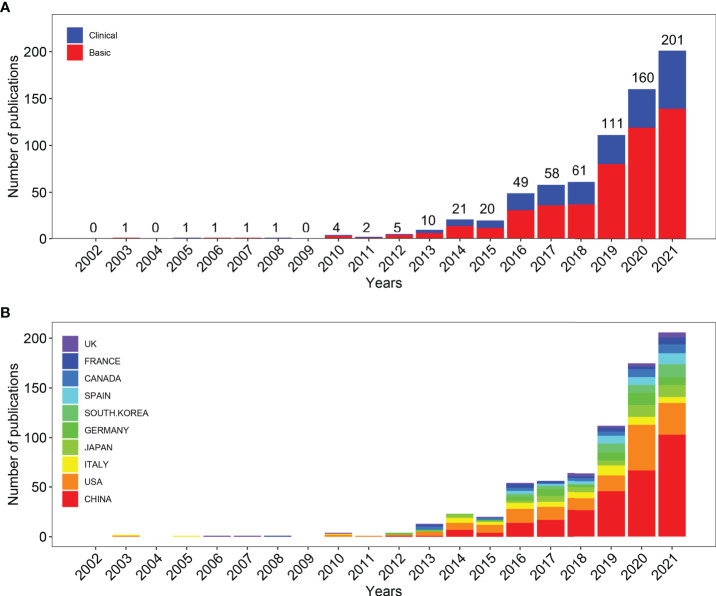
Quantity and Trends Analysis of Published Papers on the links between the gut microbiota and MAFLD between 2002 and 2021. **(A)** The number of annual research publications and research types on the links of gut microbiota and MAFLD from 2002 to 2021, export of results from Web of Sciences. **(B)** The number of annual research publications and growth trends on the links of gut microbiota and MAFLD from 2002 to 2022, export of results from the Online Analysis Platform of Literature Metrology.

Among the SCI-E of WoSCC and PubMed, a total of 1615 and 1272 articles published between 2002-2021 met the inclusion criteria, respectively. As shown in **Figure 1**, 901 and 630 literature in two databases were excluded due to their improper article types (review articles, meta-analysis, proceeding papers, or correction articles), 7 and 13 articles were excluded as non-English articles, respectively. On the basis of the defined search, 707 articles were extracted from WoSCC for the period 2002-2021. As shown in **Figure 2A**, the total number of articles published per year is listed at the top of the bar, with the colors representing the different article types. In terms of the number of publications, research on the links between gut microbiota and MAFLD can be roughly divided into two time periods. The publication trend of the early stage (2002–2011) maintained a very small quantity while the last decade (2012–2021), especially the last three years have witnessed an exponentially growing number of publications in this field, suggesting that the intricate linkage comprised in the gut microbiota and MAFLD gained worldwide attention. In addition, we employed Microsoft Excel 2022 to build a growth trend model as follows: f(x)=0.1036x^3^ - 624.07x^2^ + 1E+06x - 8E+08 (R² = 0.9854), which predicted that nearly 460 articles would be published by 2025 (**Supplementary Figure S1**). As for the type of articles, the total number of basic research and clinical research was 485 and 222, respectively. Meanwhile, basic research outnumbered clinical research under most conditions, and the growth trend of these two types of articles was the same as the growing trend of the total articles generally. In total, 629 articles (89% of WoSCC) were extracted in PubMed. The percentage of WoSCC articles that are indexed in PubMed is similar from 2002 to 2021. It is possible that PubMed did not fully cover the published articles in this field, so only the results of WoSCC searches are used for analysis in this paper, and the number of publications and research types of PubMed are shown in **Supplementary Figure S2**.

In order to figure out which country or region played the dominant role in the research of the links between the gut microbiota and MAFLD in the past two decades, the number of articles published by different countries and regions was counted on the website, the Online Analysis Platform of Bibliometrics (http://bibliometric.com/). As shown in **Figure 2B**, the bar graph presents the number of publications in the top 10 countries during the 20 years. Strikingly, the dominance of the USA in the field of gut microbiota and MAFLD among all other countries persisted until 2015, while China was positioned to be a leading role since 2016 and its publication situation maintained an increasing growth trend.

### Analysis of intercountry/region and inter-institutional cooperation

**Figure 3:**
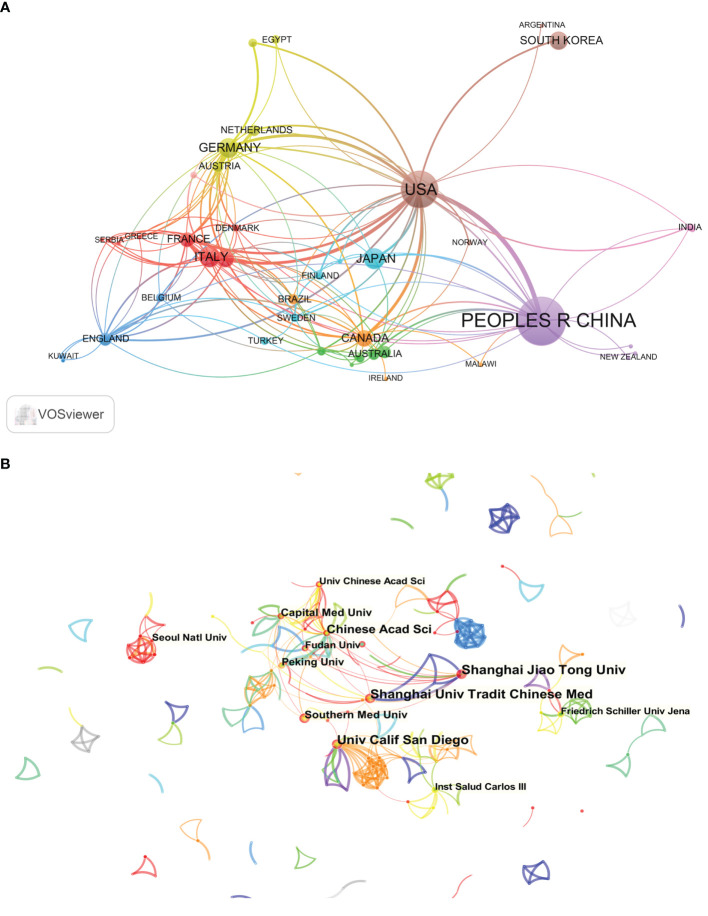
Network map of the collaboration analysis of gut microbiota and MAFLD field among countries/regions and institutions in 2002-2021. **(A)** Cooperative relationships between 53 countries/regions on the links of gut microbiota and MAFLD from 2002 to 2021. Data was exported from the Online Analysis Platform of Literature Metrology. **(B)** CiteSpace network map of institutions involved in the links between gut microbiota and MAFLD research. The top 12 most productive institutions are shown. The size of the circle represents the number of articles published by each institution and the thickness of the connecting lines indicates the degree of cooperation between institutions.

In all, the 707 articles were published by 53 countries and regions between 2002 and 2021. The Vosviewer software was applied to investigate the cooperation relation between them. As is shown in **Figure 3A**, the diagram represents the scholarly cooperation in the connection of the gut microbiota and MAFLD between countries/regions, where each circle symbolizes a country/region, and the links indicate the strength of international cooperation with each other. The size of the circle represents the number of articles published by each country/region and the thickness of the connecting lines indicates the degree of cooperation between them. The result provided evidence that the USA was the country that was the most frequently involved in international cooperation. Besides, the USA and China had the most frequent cooperation among countries/regions, and the cooperation of the USA and Italy came second.

A total number of 1237 institutes contributed to the research of the links between gut microbiota and MAFLD. In order to clarify the inter-institutional cooperation in this field, TXT format files were imported into the CiteSpace software. As is shown in **Figure 3B**, the top 12 prolific institutions are listed in the visual mapping, in which each concentric circle represents an institution, and the thickness of links indicates the strength of institutional cooperation with each other. The size of the concentric circle represents the number of articles published by each institution and the thickness of the connecting lines indicates the degree of cooperation between institutions. Amongst them, Shanghai Jiao Tong University published the most articles (22 articles) and seven institutions published exceedingly 10 articles. Eight of the top 12 most productive institutions are from China, which suggested the pivotal role of Chinese institutions in this research field.

### Analysis of co-authorship network and core author distribution

**Figure 4:**
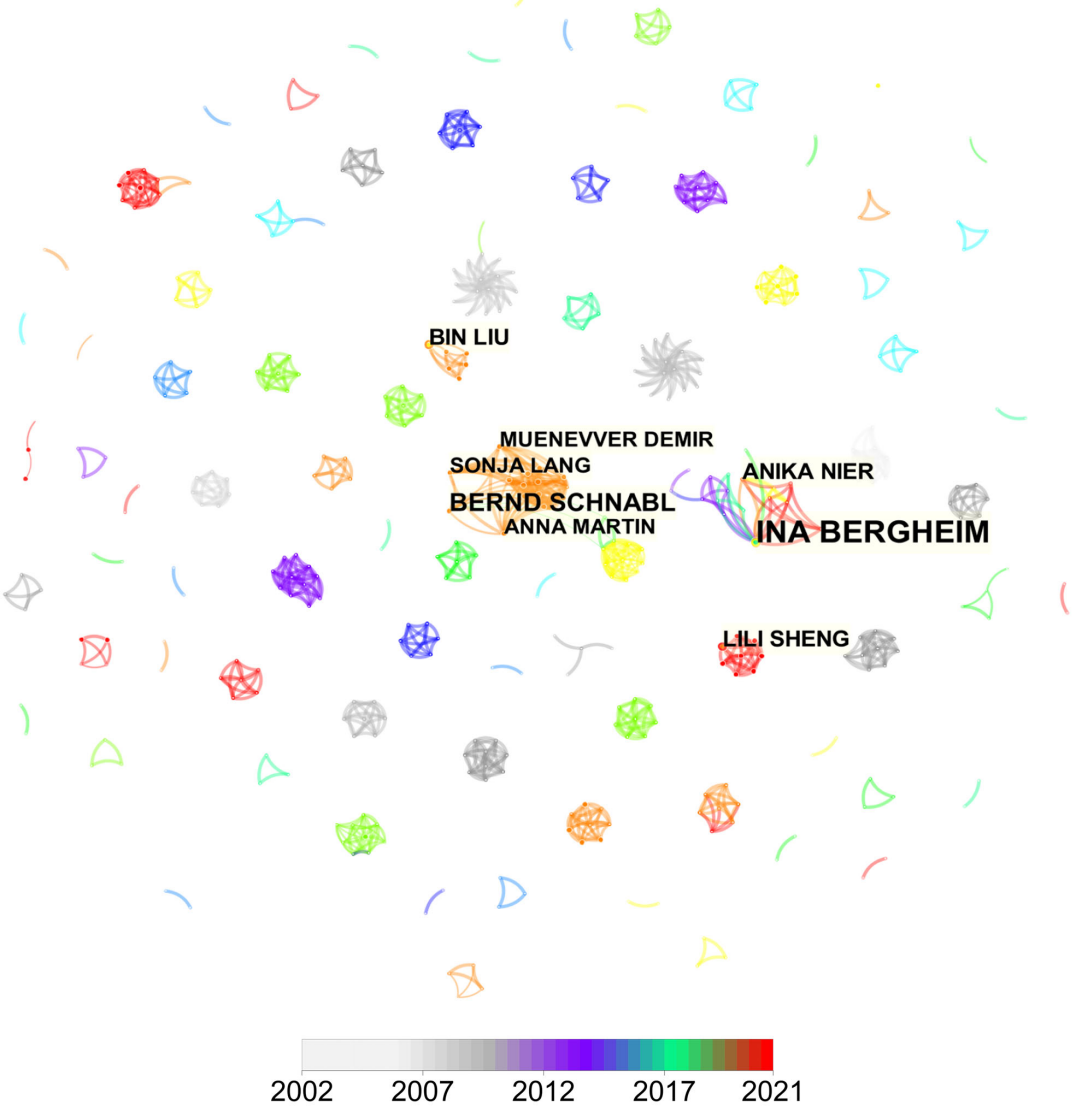
CiteSpace network of authorship in the links of gut microbiota and MAFLD research. The top 8 authors with the most publications are shown. Each circle represents an author and links between two circles mean a collaboration between each other. Font size is positively associated with the number of articles published by a certain author.

About 5347 authors made contributions to the publication outputs during the past 20 years and the top 8 most productive authors are labeled in **Figure 4**. Since there were more than 20 authors who published 4 articles, 8 authors that published more than 4 articles (5–14) were displayed. The visual mapping provides vivid information for cooperative relationships and thus helps to identify potential partners. Font size is positively associated with the number of articles published by a certain author. When it came to the connection between the gut microbiota and MAFLD scientific community, these authors were the top productive. Both Ina Bergheim (14 papers) from the University of Vienna and Bernd Schnabl (8 papers) from the University of California San Diego published over 8 articles in this field during the two decades. Notably, different from the relatively independent network in institutions, most authors especially the productive ones preferred to build steady collaborative networks.

### Analysis of journals

Over the last 20 years, 273 scholarly journals have published a total number of 707 original articles. Bibliometrics online analysis platform was used to analyze journal influence. The top 10 most-cited journals related to the links between gut microbiota and MAFLD are shown in **Table 1**, which represents those articles published in Hepatology are cited most frequently with 378 times during the past 20 years, followed by those in Scientific Reports (136), Gut (117), Nature (94), PloS One (85), Gastroenterology (79), Cell Metabolism (78), Clinical Gastroenterology and Hepatology (64), Nutrients (57) and World Journal of Gastroenterology (40). The paper published in Nature had the highest average citation per paper (47 times). In addition, half of these journals are from the UK.

**Table 1:** The top 10 most active journals that published articles in the links of gut microbiota and MAFLD research from 2002 to 2021 (sorted by total citation).

Rank	Journal title	Frequency	Total citations	Average citation per paper	Impact factor (2020)	Country	JCR
1	Hepatology	22	378	17.18	17.298	USA	Q1
2	Scientific Reports	22	136	6.18	4.379	UK	Q1
3	Gut	6	117	19.50	31.793	UK	Q1
4	Nature	2	94	47.00	69.504	UK	Q1
5	PloS One	17	85	5.00	3.240	USA	Q2
6	Gastroenterology	8	79	9.88	33.883	UK	Q1
7	Cell Metabolism	5	78	15.6	31.373	USA	Q1
8	Clinical Gastroenterology and Hepatology	2	64	32.00	13.576	UK	Q1
9	Nutrients	38	57	1.50	6.706	Swit	Q2
10	World Journal of Gastroenterology	11	40	3.64	5.21	CHN	Q2

### Analysis of number of citations

The number of citations is an important indicator of the impact of an article in a research area. The number of citations of these 707 articles was counted and ranked, and the top 10 are shown in **Table 2**. The most cited article was published in Nature by Jorge Henao-Mejia et al. of Yale University in 2012 (24), which has been cited 1586 times. This paper showed that the NLRP6 and NLRP3 inflammasomes negatively regulate MAFLD/NASH progression and metabolic syndrome by modulating the gut microbiota, highlighting the central role of the microbiota in the pathogenesis of systemic auto-inflammation and metabolic disorders. The second and third articles were published in PNAS and Hepatology, with 777 and 638 citations, respectively (25, 26). Both supported the idea that intestinal bacteria play an important role in the development of insulin resistance and MAFLD. The 10^th^ place was published in Journal of Clinical Investigation in 2015, with 389 citations (27).

**Table 2:** The top 10 most cited articles from 707 retrieved list in the links of gut microbiota and MAFLD research from 2002 to 2021 (sorted by cited frequency).

Rank	Title	First author	Journal	Year	Cited frequency	DOI
1	Inflammasome-mediated dysbiosis regulates progression of NAFLD and obesity	Henao-Mejia, J	Nature	2012	1586	10.1038/nature10809
2	Metabolic profiling reveals a contribution of gut microbiota to fatty liver phenotype in insulin-resistant mice	Dumas, ME	PNAS	2006	777	10.1073/pnas.0601056103
3	The Severity of Nonalcoholic Fatty Liver Disease Is Associated With Gut Dysbiosis and Shift in the Metabolic Function of the Gut Microbiota	Boursier, J	Hepatology	2016	649	10.1002/hep.28356
4	Probiotics and antibodies to TNF inhibit inflammatory activity and improve nonalcoholic fatty liver disease	Li, ZP	Hepatology	2003	638	10.1053/jhep.2003.50048
5	Intestinal microbiota determines development of non-alcoholic fatty liver disease in mice	Le Roy, T	Gut	2013	607	10.1136/gutjnl-2012-303816
6	Intestinal Microbiota in Patients With Nonalcoholic Fatty Liver Disease	Mouzaki, M	Hepatology	2013	459	10.1002/hep.26319
7	Gut Microbiome-Based Metagenomic Signature for Non-invasive Detection of Advanced Fibrosis in Human Nonalcoholic Fatty Liver Disease	Loomba, R	Cell Metabolism	2017	459	10.1016/j.cmet.2017.04.001
8	Association Between Composition of the Human Gastrointestinal Microbiome and Development of Fatty Liver With Choline Deficiency	Spencer, MD	Gastroenterology	2011	423	10.1053/j.gastro.2010.11.049
9	Fecal Microbiome and Volatile Organic Compound Metabolome in Obese Humans With Nonalcoholic Fatty Liver Disease	Raman, M	Clinical Gastroenterology and Hepatology	2013	416	10.1016/j.cgh.2013.02.015
10	Intestinal farnesoid X receptor signaling promotes nonalcoholic fatty liver disease	Jiang, CT	Journal of Clinical Investigation	2015	389	10.1172/JCI76738

**Figure 5:**
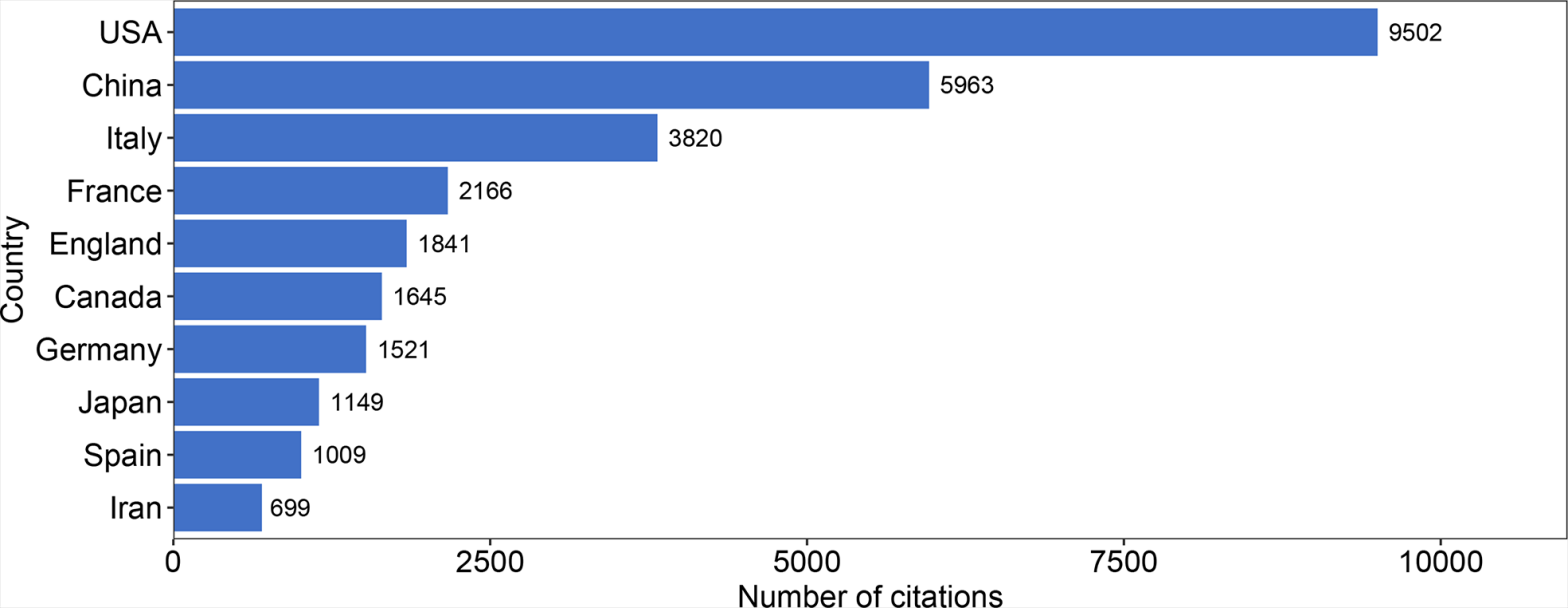
Bar graph of the total number of citations from 707 retrieved articles among countries in 2002-2021. The top 10 countries with the highest total number of citations are shown. Each bar represents a country, and the length is positively correlated with the total number of citations.

We also summarized the total number of citations of all articles published by each country and presented the top ten countries according to the total number of citations in **Figure 5**. During this 20-year period, the total number of citations of articles published in the USA ranked first with 9502. China and Italy ranked second and third with 5963 and 3820. The 10^th^ place is Iran with a total of 699 citations. Surprisingly, we found that the total number of citations of articles from seven countries (fourth to tenth) is 10,030, which is almost equal to the first ranked country, the USA.

Combining the total number of publications and the total number of citations in each country, the average number of citations per article in each country can be obtained (total number of citations of each country/total number of publications of each country). The top ten countries sorted by average number of citations are shown in **Table 3**. The top three countries with the highest average number of citations per article were the UK (average of 96.9 citations per article), France (86.6), and Italy (73.5), and each article from these countries is cited more than 70 times on average. In terms of the 19 articles from the UK, four articles were cited more than 100 times, with the most cited being a paper by Marc-Emmanuel Dumas et al. with 777 citations, ranking second in the world (25). The second most cited study in the UK was from Maitreyi Raman et al. with 416 citations, ranking 9^th^ in the world (28).

**Table 3:** The top 10 countries with the highest average number of citations per article from 707 retrieved list in the links of gut microbiota and MAFLD research from 2002 to 2021 (sorted by the average number of citations).

Rank	Country	Number of publications	Total number of citations	Average number of citations
1	UK	19	1841	96.9
2	France	25	2166	86.6
3	Italy	52	3820	73.5
4	USA	159	9502	59.8
5	India	7	368	52.6
6	Canada	33	1645	49.8
7	Iran	16	699	43.7
8	Finland	8	267	33.4
9	Switzerland	8	261	32.6
10	Germany	47	1521	32.4

### Analysis of document co-citation and clustered network

**Figure 6:**
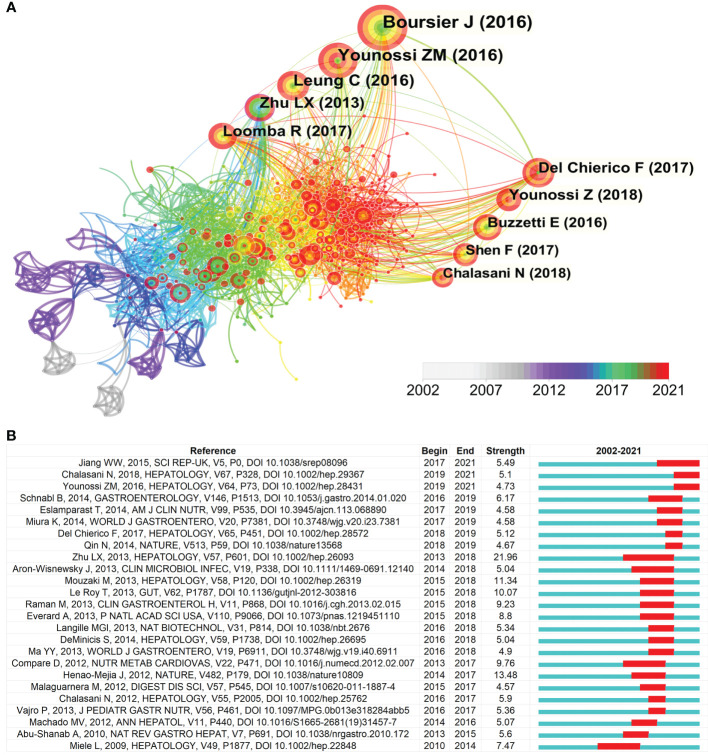
Reference co-citation network analysis of publications on the links between the gut microbiota and MAFLD between 2002 and 2021. **(A)** CiteSpace co-citation map of 20616 references on the links of gut microbiota and MAFLD research, filter option showing the largest connected component only. Each node represents a reference and the links between nodes mean these articles were cited as references in the same article within the retrieved 707 articles. The size of the node is positively related to the frequency of citation and line thickness means the correlation with the co-cited papers. The color represents the year of publication as shown in the color bar. **(B)** Reference with the strongest burst strength of the 707 citing articles on the links of gut microbiota and MAFLD research between 2002 and 2021. Reference marked in red indicates a sudden increase in usage frequency of this reference during that period. Blue represents a relatively unpopular time period.

Co-cited references are those cited by more than one article of the 707 extracted list. Map of co-citation reference in CiteSpace on the links between the gut microbiota and MAFLD were presented in **Figure 6A**. Each node represents a reference and the links between nodes mean these articles are cited as references in the same article within the retrieved 707 ones. The size of the node is positively related to the frequency of citation and line thickness means the correlation with the co-cited papers. Additionally, according to the color bar, the redder nodes represent that these papers have been frequently cited in recent years while the greyer ones represent those references cited in earlier years. The top ten references of 707 retrieved papers sorted by frequency of citations are shown in **Table 4**.

**Table 4:** The top 10 high cited references of 707 retrieved articles in the links of gut microbiota and MAFLD research from 2002 to 2021.

Rank	Title	First author	Journal	Year	Cited frequency	Centrality	DOI
1	The severity of nonalcoholic fatty liver disease is associated with gut dysbiosis and shift in the metabolic function of the gut microbiota	Boursier J	Hepatology	2016	116	0.08	10.1002/hep.28356
2	Global epidemiology of nonalcoholic fatty liver disease-Meta-analytic assessment of prevalence, incidence, and outcomes	Younossi ZM	Hepatology	2016	80	0.02	10.1002/hep.28431
3	The role of the gut microbiota in NAFLD	Leung C	Nat Rev Gastro Hepat	2016	75	0.03	10.1038/nrgastro.2016.85
4	Characterization of gut microbiomes in nonalcoholic steatohepatitis (NASH) patients: a connection between endogenous alcohol and NASH	Zhu LX	Hepatology	2013	58	0.12	10.1002/hep.26093
5	Gut Microbiome-Based Metagenomic Signature for Non-invasive Detection of Advanced Fibrosis in Human Nonalcoholic Fatty Liver Disease	Loomba R	Cell Metab	2017	55	0.06	10.1016/j.cmet.2017.04.001
6	Gut microbiota profiling of pediatric nonalcoholic fatty liver disease and obese patients unveiled by an integrated meta-omics-based approach	Del Chierico F	Hepatology	2017	54	0.04	10.1002/hep.28572
7	Global burden of NAFLD and NASH: trends, predictions, risk factors and prevention	Younossi Z	Nat Rev Gastro Hepat	2018	52	0.01	10.1038/nrgastro.2017.109
8	The multiple-hit pathogenesis of non-alcoholic fatty liver disease (NAFLD)	Buzzetti E	Metabolism	2016	47	0.02	10.1016/j.metabol.2015.12.012
9	The diagnosis and management of nonalcoholic fatty liver disease: Practice guidance from the American Association for the Study of Liver Diseases	Chalasani N	Hepatology	2018	38	0.04	10.1002/hep.29367
10	Gut microbiota dysbiosis in patients with non-alcoholic fatty liver disease.	Shen F	Hepatob Pancreat Dis	2017	38	0.02	10.1016/S1499-3872(17)60019-5

Results showed that the highest-ranking cited reference was clinical research published by Hepatology in 2016. 57 patients diagnosed with NAFLD were enrolled and the research found out that Bacteroides was independently associated with NASH and Ruminococcus was closely associated with significant fibrosis (26). The second-ranked reference was a meta-analysis review, which was also published by Hepatology in 2016. Researchers retrieved and analyzed a total number of 8,515,431 patients from the published studies from 1989 to 2015 in order to determine the prevalence, incidence, risk factors, and long-term outcomes of patients with NAFLD (29). The third-ranked reference was a review issued by Nature Review Gastroenterology & hepatology in 2016, exploring the links between NAFLD, metabolic syndrome, dysbiosis, poor diet, and gut health (16). The first and third articles both emphasized inter-kingdom signaling, which means the interaction between gut bacteria and the host system, and suggested the possibility of applying gut microbiota as a clinical marker for diagnosing, grading, and treating NAFLD. The second reference provided detailed epidemiological information on NAFLD to demonstrate its importance. Notably, the highest centrality is an article published by Lixin Zhu et al. in Hepatology, which suggested distinct composition of the gut microbiome among obese and healthy controls could offer a target for intervention or a marker for disease (30). In general, the most cited reference listed in **Table 4** made great contributions to illustrate the connection between the gut microbiota and MAFLD scientific community and could be regarded as the most approved in this field.

The references burst into this field were also analyzed which helped to find out research hotspots, that referred to the high frequency of presence of these references during a period (31). **Figure 6B** shows the top 25 references burst as citations in the connection of the gut microbiota and MAFLD scientific community during the period of 2002-2021. The blue line indicates the time range from 2002 to 2021 and the red line indicates the period that the burst references maintain. The latest references burst appearing in 2017 and lasting till the end of 2021 was the clinical research published by Scientific Reports in 2015 (32). 53 NAFLD patients and 32 healthy subjects were recruited, and researchers found that aside from dysbiosis, gut microbiota-mediated inflammation of the intestinal mucosa and the related impairment in mucosal immune function also played an important role in the pathogenesis of NAFLD. The second reference in **Figure 6B** was a review published by Hepatology in 2018, burst in 2019, and lasted till the end of 2021 (33). This review summarized the diagnosis and management of NAFLD. Among these reference bursts, the highest strength was 21.96 from clinical research published by Hepatology in 2013, which burst in 2013 and finished in 2018 (30). NASH, obese, and healthy children were recruited in the study, and an increased abundance of alcohol-producing bacteria and elevated blood-ethanol concentration were found in NASH patients, which could offer a target for intervention or a marker for the disease.

**Figure 7:**
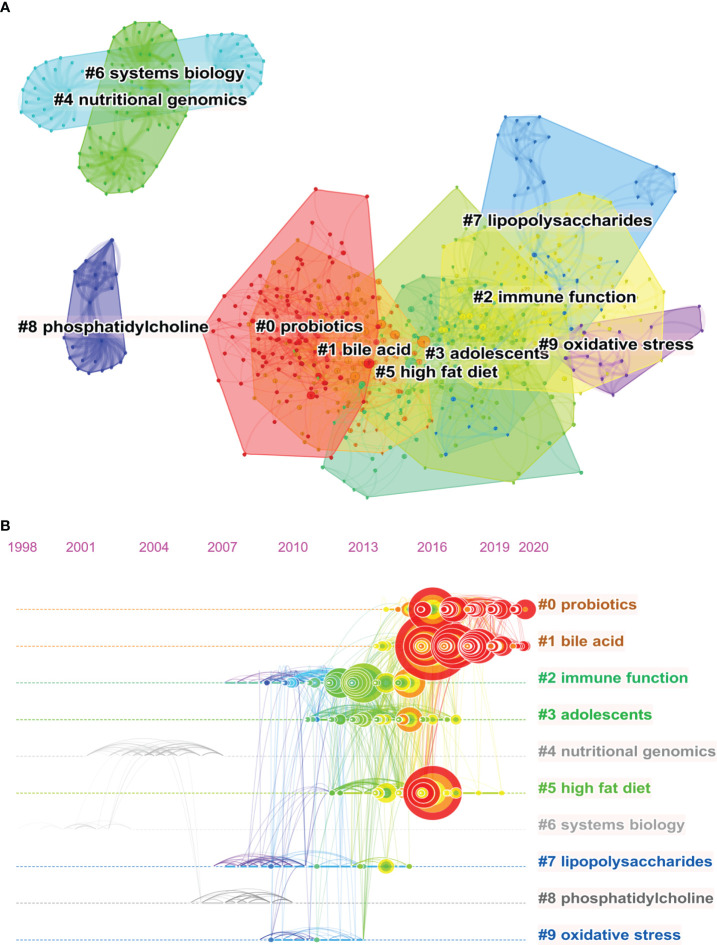
Analysis of Co-occurring Keywords of publications on the links between the gut microbiota and MAFLD between 2002 and 2021. **(A)** Clustered networks of co-citation status of the investigated reference and the 707 citing articles *via* CiteSpace. The top 10 largest clusters of citing articles on the links between gut microbiota and MAFLD research are shown. **(B)** A timeline view of the top 10 largest clusters of citing articles on the links between gut microbiota and MAFLD research. Right side = cluster labels.

The map of co-citation clustered according to keywords integrated from the references of 707 retrieved articles by CiteSpace is shown in **Figure 7A**. The analysis of co-citation clusters showed the most relevant terms on the links of the gut microbiota and MAFLD research by the approach of hierarchical cluster labels, which includes #0 probiotics, #1 bile acid, #2 immune function, #3 adolescents, #4 nutritional genomics, #5 high fat diet, #6 systems biology, #7 lipopolysaccharides, #8 phosphatidylcholine and #9 oxidative stress. The number of cluster labels is reversely correlated with the number of articles that each cluster contains. In other words, the cluster marked as #0 contains the largest number of papers among the 20616 co-cited references. A summary of clusters is listed in **Supplementary Table S1**.

### Analysis of research trend and burst detection with keywords

**Figure 8:**
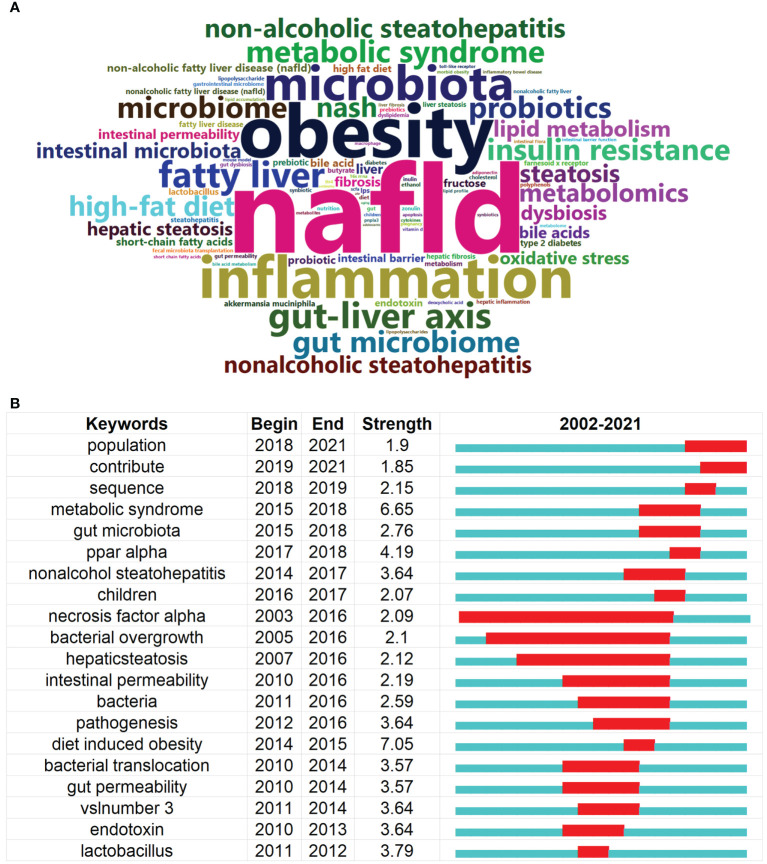
Analysis of Keywords and Burst Detection of publications on the links between the gut microbiota and MAFLD between 2002 and 2021. **(A)** Word cloud of the top 100 high-frequency keywords on the links of gut microbiota and MAFLD research *via* the R package bibliometrix **(B)** Keywords with the strongest burst strength of the 707 citing articles on the links of gut microbiota and MAFLD research between 2002 and 2021. Keyword marked in red indicates a sudden increase in usage frequency of this keyword during that period. Blue represents a relatively unpopular time period.

With the aim of clearly describing the shift of hotspots in the links between the gut microbiota and MAFLD in the last two decades, a timeline view is displayed in **Figure 7B**. As shown in the illustration, each circle represents a main cited paper in a certain cluster and the citation tree-rings of different sizes on the timeline represent citation rates. Large nodes are highly cited or have citation bursts in each time slice. The co-citation with the keyword of immune function was prevalent began in 2007 and ended in 2016. The cluster of adolescents and high fat diet were hotspots that started in almost 2012 and ended in 2016. The latest hotspots were bile acid and probiotics, which emerged in 2013 and kept till now. Overall, the center of research in this field seems to have been transferred from immune function, adolescents, and high fat diet to bile acid and probiotics. A word cloud as shown in **Figure 8A** represents the top 100 high-frequency keywords on the links between gut microbiota and MAFLD research. Font size is positively associated with frequency. After deleting the ones with little real significance, obesity, inflammation, gut-liver axis, and metabolic syndrome were the keywords with high frequency.

Keyword burst detection is another approach that helps to find out research hotspots. **Figure 8B** shows the top 20 references with the strongest keywords bursts in the connection between the gut microbiota and MAFLD scientific community during the period 2002-2021. The blue line indicates the time range from 2002 to 2021 and the red line indicates the period that the burst keywords maintain. Since none of the keywords burst with real significance which lasted till the end of 2021 were found, the keywords with the highest strength were listed. Amongst them, the keyword burst, diet induced obesity, began in 2014 and lasted 1 year with the highest strength of 7.05, which was a precursor of MAFLD. The second strength 6.65 belonged to metabolic syndrome, beginning in 2015 and ending in 2018, that was associated with the concept of MAFLD. The third keyword burst was ppar alpha with a strength of 4.19 during 2017-2018. It was a nuclear receptor expressed in tissues that plays a central role in metabolism, which is related to lipid accumulation in MAFLD. Lactobacillus was the fourth keyword burst with a strength of 3.79 between 2011-2012, and it was a species of gut microbiota that could attenuate the progression of MAFLD.

## Discussion

In this bibliometric analysis study, we found 707 articles on the connection between gut microbiota and MAFLD research from 2002 to 2021 in SCI-E of WoSCC. The overall publication number has been increasing consistently, and even presented a blowout type growth after 2015. With the approach of the online bibliometric analysis platform and CiteSpace software, we analyzed publication trends about the connection between gut microbiota and MAFLD by timeline, research type, and countries/regions. The representative clusters of cited articles were probiotics and bile acid. Diet induced obesity, metabolic syndrome, ppar alpha, and lactobacillus were the keywords burst with the highest strength in the last two decades. Thus, with the help of this bibliometrics analysis, researchers interested in this field can easily have a general understanding and quickly get the latest research hotspots.

Between 2002 and 2011, fewer articles were published in the gut microbiota and MAFLD fields. However, the number of papers has been increasing since 2011 and has even shown exponential growth in recent years, indicating that the research of gut microbiota and MAFLD has attracted the attention of researchers worldwide. As MAFLD is the most common liver disease in this generation and new tools to investigate gut microbiota have been invented, gut microbiota and MAFLD will continue to be a trend for a long time, and the number of papers will continue to soar.

During the last two decades, the proportion of basic research is around 60%-70%, almost double that of clinical research articles. Amongst them, most of the clinical studies are correlation analysis of gut microbiota and MAFLD. Mara P H van Trijp et al. reported that minor changes in the composition of the gut microbiota during a 12-week wheat intervention correlate with liver fat in overweight and obese adults (34). Yingyue Tang et al. showed NAFLD affect hepatic lipid accumulation and correlate with gut dysbiosis (35). However, only a few articles regarded gut microbiota as a diagnostic marker or a therapeutic target. Tien S Dong et al. suggested that microbial profiles can be used as a non-invasive marker for advanced fibrosis, a severe complication of MAFLD (36). The first reason is that the technology of intestinal bacteria sequencing is mostly kept in basic animal research, and the detection, diagnosis, and treatment of intestinal microbiota have not been popularized in the clinical environment. On the other hand, the most common animal model of gut microbiota is that of mice, whose gut microbiota is not the same as humans (37). Finally, the requirements of clinical research on patients’ safety are much higher, and the follow-up and management of patients are also much more difficult than that of experimental animals. However, because of the differences between animal and human species, the results of animal studies are not entirely applicable to humans. Therefore, more clinical studies are needed to advance this field.

China and the USA played leading roles in the field of gut microbiota and MAFLD research in recent years, as evidenced by the number of papers published in this field. Before 2016, the USA was the biggest contributor to the sector. However, since 2016, the number of articles published in China has increased year by year and surpassed that in the USA, and notably, the number of publications in China accounted for half of the global total in 2021. There may be three main reasons. First, China has a large number of researchers in the gut microbiota and MAFLD field. Secondly, the scientific research capability of Chinese researchers has also risen greatly. At the level of cooperation, the USA had the strongest ties with other countries, and next comes China and Germany. Among them, the cooperation between China and the USA was the closest, the second was the USA and Italy, and these showed the central position of the USA in the world’s academic activities. At the same time, research cooperation between the USA and China, and other countries are not yet very adequate, and we presume that the domestic scientific cooperation in each country has been adequate enough. In the last several years, covid-19 pandemic affected international scientific collaborations.

In the statistics of the number of publications between institutions, four of the top five were from China, and two were from Shanghai. These results demonstrated the leading role played by Chinese institutions in the field of gut microbiota and MAFLD, especially the academic institutions in Shanghai, for example, Shanghai Jiao Tong University and Shanghai University of Traditional Chinese Medicine. The reason is, first, the number of academic institutions in Shanghai gives it a great basis for academic production. Secondly, the comprehensive strength of many academic institutions in Shanghai ranks near the top in the world. In institutional cooperation, most institutions tended to cooperate with domestic institutions and less with foreign institutions.

For the authors that contributed to the links between gut microbiota and MAFLD in the past 20 years, Ina Bergheim from the Department of Nutritional Sciences of the University of Vienna published 14 articles in this field. Univ.-Prof. Dr. Ina Bergheim was an expert in NASH and gut bacteria, especially in the gut-liver axis. Bernd Schnabl from the University of California San Diego published 8 articles. The main interest of Dr. Bernd Schnabl was intestinal microbiome, metagenome associated with liver disease, and contribution of microbial products and metabolites to liver disease.

In the top 10 most active journals that published articles on the links between gut microbiota and MAFLD research, the journal Hepatology possessed the most total citations (378). Hepatology is the top journal in the field of the liver containing all aspects of liver structure, function, and disease. Besides, papers published in Nature occupied the highest average citation (47), which was probably because Nature was a well-known authoritative journal around the world. Of the 10 journals, five were from the UK, three were from the USA, and the remaining two were from Switzerland and China, reflecting the fact that the UK primarily provided a communication platform for research on the connection between gut microbiota and MAFLD.

Among the top 10 articles with the highest number of citations, clinical research and basic research each accounted for half of the total. Combining the publication dates of these articles, it is easy to find that most of the basic-type articles were published earlier (two before 2010, three before 2015), while the clinical-type articles were published later (three published in 2010-2015, two after 2015), which probably because the mechanism was first explored in experimental animals and then validated and applied to patients. Among these 10 articles, the two most cited articles are both basic research, which focused on the role of microbiota on insulin resistance and metabolic syndrome in the development of MAFLD, indicating that the relationship between microbiota and systemic metabolism is now a hot spot of research in the field of gut microbiota and MAFLD (24, 25). The focus of these 5 clinical-type papers evolved from the relationship between gut microbes and the presence of MAFLD to the severity of MAFLD or related liver fibrosis, reflecting the deepening of the involvement of gut microbes in the diagnosis and treatment of MAFLD over these 20 years (26, 28, 38–40).

For the total number of citations of each country, the USA and China were undoubtedly the two most influential countries in the field of gut microbiota and MAFLD during this 20-year period. The very key reason is that the USA and China are also ranked second and first in the world, respectively, in terms of the number of articles published. But things change when the average number of citations per article is considered. The countries with the highest average number of citations per article were the UK, France, and Italy, indicating the high average quality of articles from these three countries. The small account and high quality are the reason why the UK has the highest average number of citations per article in the world. The average number of citations per article in China ranked 16th, which indicates that although China leads the world in the number of published articles, the average quality needs to be improved. In fact, there were 4 articles from China with more than 200 citations and 13 articles with more than 100 citations, which indicates there are many high-level articles recognized by peers (27, 32, 41, 42). However, there were more articles with a low number of citations, with 79 articles having no more than 5 citations.

Emerging trends and hotspots were analyzed by CiteSpace. Keyword burst is considered an important indicator of trends and hotspots. As shown in **Figure 8B**, the top 20 keywords bursts with the strongest citation were listed, revealing potential hotspots on the links between gut microbiota and MAFLD over the last 20 years. Interestingly, most bursts of these keywords began before 2012 but didn’t continue till 2016, indicating that such research trends represented that generation of this field. As none of the keywords burst with real significance which lasted till the end of 2021 were discovered, the keywords with strong strength were discussed here. Among them, diet induced obesity, a keyword burst that began in 2014 and lasted until the end of 2015 with the highest strength of 7.05. Diet-induced obesity is a main cause of MAFLD and resulted in aberrant accumulation of lipids in hepatocytes and is highly correlated with other metabolic disorders, including insulin resistance, and type 2 diabetes. Overnutrition and consumption of highly processed foods are the characteristics of an improper diet.

The second keyword burst was metabolic syndrome, beginning in 2015 and lasting till the end of 2018 with a strength of 6.65 ranked second highest. Metabolic syndrome is a cluster of metabolic abnormalities that identifies people at risk of diabetes mellitus and cardiovascular disease, whereas NAFLD is considered a manifestation of metabolic syndrome in the liver (43, 44). After the renaming, the definition of MAFLD is defined as a hepatic steatosis entity in addition to the presence of evidence of metabolic dysfunction, highlighting the vital role of the metabolic factor in the etiology (45).

The third was ppar alpha, which was a nuclear receptor expressed in tissues with a high oxidative activity that played a central role in metabolism. This keyword burst began in 2017 and ended in 2018 with a strength of 4.19. PPAR alpha is crucial for whole-body fatty acid homeostasis and is protective against MAFLD and the depletion of PPAR alpha in the liver leads to lipid accumulation in hepatocytes. These findings paved the way for the potential of hepatocyte PPAR alpha as a drug target for MAFLD (46).

The fourth keyword burst was lactobacillus with a strength of 3.79, beginning in 2011 and ending in 2012. Mounting preclinical evidence broadly indicated that lactobacillus could ameliorate the progression of MAFLD in many ways by improving the intestinal microecology and mucosal barrier, and by modulating the tryptophan pathway. In this sense, lactobacillus could be used as potential probiotic candidates to prevent MAFLD (47, 48).

The study also has limitations. First, the current version of CiteSpace can only analyze and visualize co-citation map of data extracted from SCI-E in WoSCC database, while data from other databases such as PubMed and Embase cannot support co-citation analysis. Second, due to search conditions, literature types, and language constraints, our search strategy may not include all relevant articles. Moreover, although the number of articles published in the field has surged in recent years, the overall number remains relatively small. Therefore, the results of our analysis based on current literature may be slightly biased. In future research, we should pay attention to and avoid these limitations as much as possible.

## Conclusion

In this study, we investigated the links between gut microbiota and MAFLD field with the method of CiteSpace, Vosviewer, and the Online Analysis Platform of Literature Metrology. The number of publications increased dramatically in the past decade and especially became exponential growth in the last 3 years, indicating researchers were paying more attention to the field. In addition, the current focus on probiotics and bile acid is critical for the mechanism of interaction between gut microbiota and MAFLD and for the exploration of novel effective treatments of MAFLD. This bibliometric study defines the overall prospects in the field of the links between gut microbiota and MAFLD and provides valuable information for ongoing studies.

## Data availability statement

The raw data supporting the conclusions of this article will be made available by the authors, without undue reservation.

## Author contributions

LY and PG raised the conception of the study and designed the study. YL screened articles and wrote the original manuscript. YZ and LW conducted the CiteSpace and VOSviewer analysis. XL, MM, SY and LZ helped interpreted the data for the work. LY, PG, YZ, LW, XL, MM, YJ and WY revised the manuscript and edited critically. All authors contributed to the article and approved the submitted version.

## Funding

This study was funded by the National Natural Science Foundation of China (No. 82270916), Shanghai Hospital Development Center (No. SHDC2020CR2055B), Science and Technology Commission of Shanghai Municipality (No. 20410760500), National key research and development program (No. 2018YFC201803), Key Specialty Construction Project of Pudong Health and Family Planning Commission of Shanghai (No. PWZXQ2017-06), Shanghai Municipal Key Clinical Specialty (No. shslczdzk03601), Innovation Program of Shanghai Municipal Education Commission (No. 2019-01-07-00-01-E00074) and Shanghai Engineering Research Center of Peri-operative Organ Support and Function Preservation (20DZ2254200).

## Acknowledgments

We thank ‘Clarivate Analytics—Web of Science’ to provide data access. We also would like to express our appreciation to CiteSpace and VOSviewer to help results analysis.

## Conflict of interest

The authors declare that the research was conducted in the absence of any commercial or financial relationships that could be construed as a potential conflict of interest.

## Publisher’s note

All claims expressed in this article are solely those of the authors and do not necessarily represent those of their affiliated organizations, or those of the publisher, the editors and the reviewers. Any product that may be evaluated in this article, or claim that may be made by its manufacturer, is not guaranteed or endorsed by the publisher.
