# Supplementary material for: Emerging trends and hotspots in the links between the gut microbiota and MAFLD from 2002 to 2021: A bibliometric analysis
Source: Front Endocrinol (Lausanne). 2022 Oct 13;13:990953. doi: 10.3389/fendo.2022.990953 (PMC9624192; doi:10.3389/fendo.2022.990953)
Supplement: Supplementary file 1 [file DataSheet_1.docx]

Supplementary Material


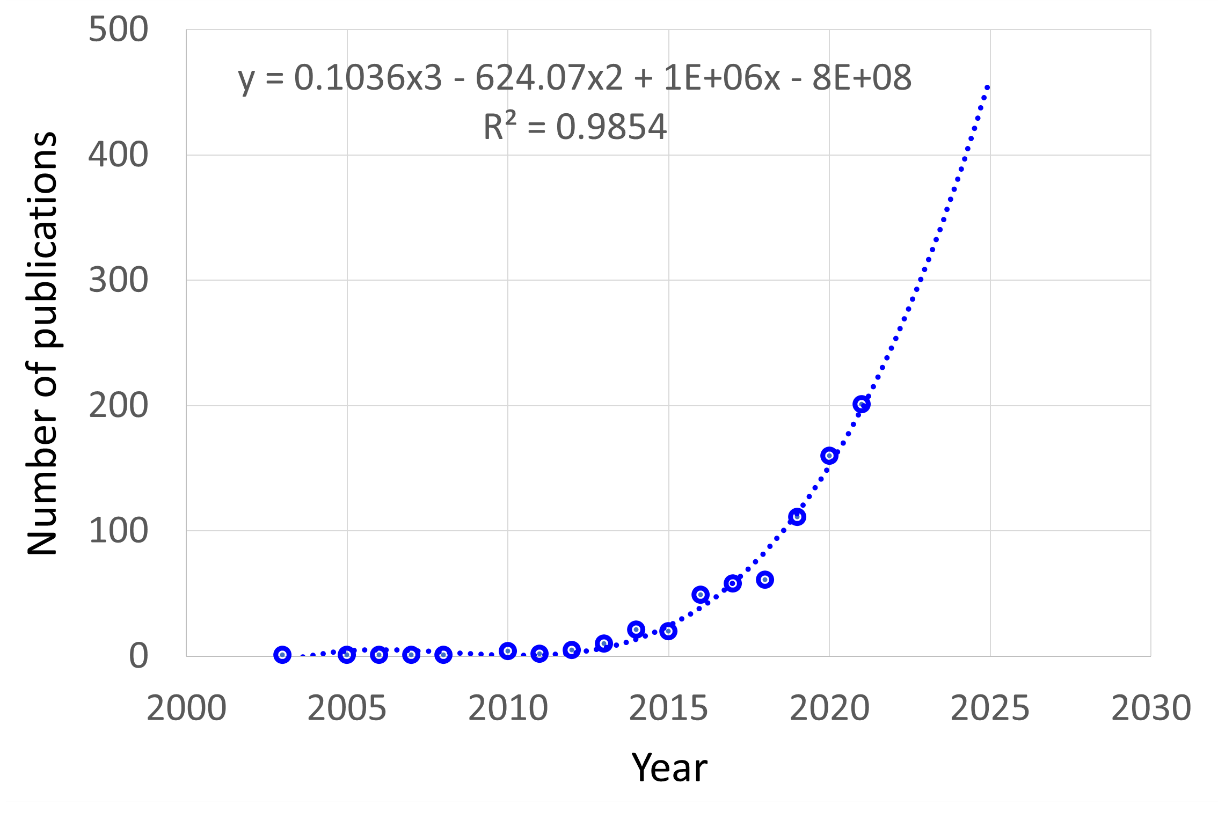


**Supplementary Figure 1**. The output of publications and growth prediction of research on the links between gut microbiota and MAFLD. The number of publications from 2002 to 2021 is presented by scatters. The dashed line represents the predicted curve, R^2^=0.9854.


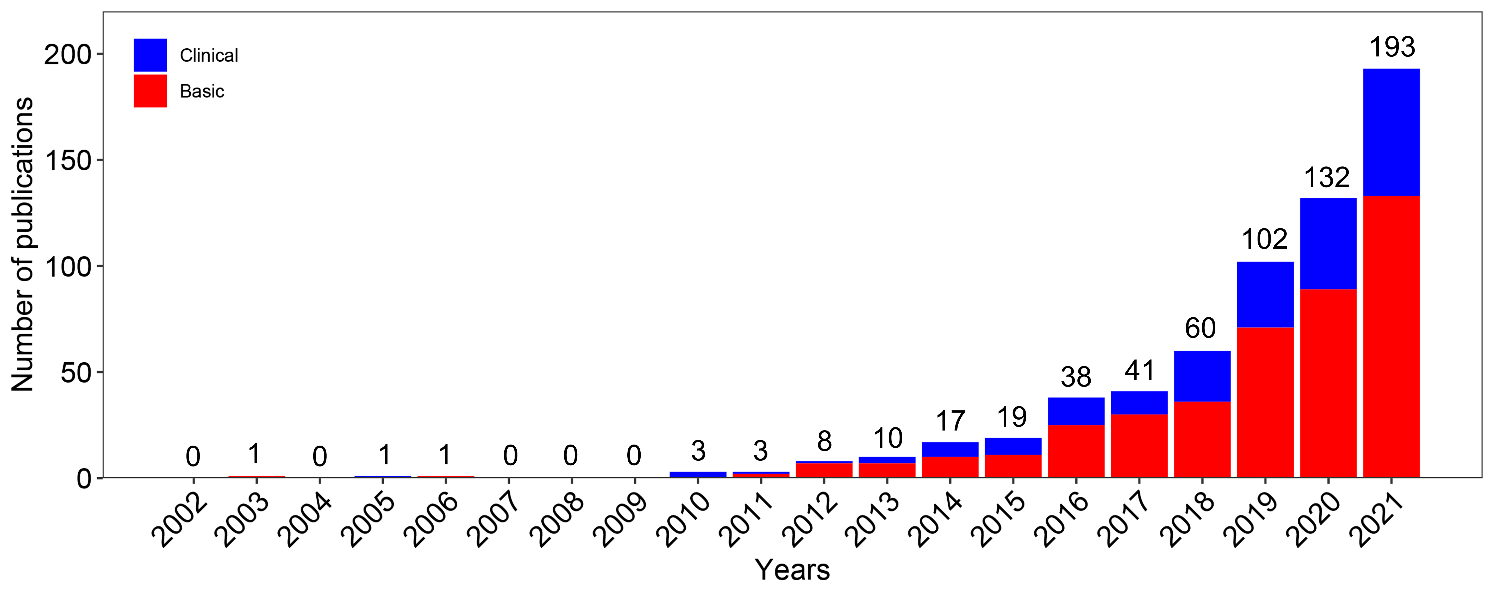


**Supplementary Figure 2**. The number of annual research publications and research types on the links of gut microbiota and MAFLD from 2002 to 2021, export of results from PubMed.

| **Cluster ID** | **Top Term** | **Size** | **Silhouette** |
| --- | --- | --- | --- |
| 0 | probiotics | 122 | 0.78 |
| 1 | bile acid | 121 | 0.843 |
| 2 | immune function | 103 | 0.818 |
| 3 | adolescents | 96 | 0.828 |
| 4 | nutritional genomics | 65 | 0.985 |
| 5 | high fat diet | 61 | 0.844 |
| 6 | systems biology | 49 | 0.985 |
| 7 | lipopolysaccharides | 41 | 0.934 |
| 8 | phosphatidylcholine | 26 | 0.995 |
| 9 | oxidative stress | 19 | 0.984 |

**Supplementary Table 1.** Summary of the top 10 largest clusters of citing articles on the link between gut microbiota and MAFLD research (Silhouette value >0.5 means the clustering results are reliable).
